# Back and neck pain and function in females with adolescent idiopathic scoliosis: A follow-up at least 23 years after conservative treatment with a Milwaukee brace

**DOI:** 10.1371/journal.pone.0189358

**Published:** 2017-12-11

**Authors:** Ewa Misterska, Jakub Głowacki, Adam Okręt, Maria Laurentowska, Maciej Głowacki

**Affiliations:** 1 Department of Pedagogy and Psychology, University of Security, Poznan, Poland; 2 HCP Medical Center, Poznan, Poland; 3 Department of Physiology, Poznan University of Physical Education, Poznan, Poland; 4 Department of Pediatric Orthopaedics and Traumatology, Poznan University of Medical Sciences, Poznan, Poland; University of Illinois at Urbana-Champaign, UNITED STATES

## Abstract

We aimed to explore the long-term outcomes of back and neck pain and functionality in adult females with adolescent idiopathic scoliosis (AIS), who had been treated with a Milwaukee brace, in a follow-up study a minimum of 23 years after the completion of the treatment, using radiological, clinical and socio-demographical data. Thirty AIS patients (scoliosis group–SG), were included in the study based on an extensive search of Pediatric Orthopedics and Traumatology Clinic charts. All treatments were successfully completed between 1974 and 1990. In all cases, scoliosis had not been detected before the age of 10 and was not combined with any major spinal deformities at the time when the brace treatment was implemented. In those patients, the Risser sign 4 and minimum two years post-menarche was defined as a maturity, after that time the brace treatment was completed. Patients were excluded from the study if they, at the time of the follow-up examinations, suffered from any other disease leading to trunk deformity. Forty patients met the criteria for inclusion, but due to change some personal details, not all of them were contacted. Finally, 30 women returned for a follow-up evaluation. Patients’ follow-up period was mean 27.77 yrs. ± SD 3.30 (range 23–35). Curvature change from the end of the treatment until the present day was mean 9.1 degrees ± SD 7.64 (range 0–27). A control group of 42 healthy females (healthy controls group—HG) matching the age profile of the patient group was randomly selected for comparative purposes.Both SG and HG completed the Polish versions of the Revised Oswestry Lower Back Pain Disability Index (RODI), the Rolland-Morris Questionnaire (RMQ), the Quebec Back Pain Disability Scale (QDS), the Neck Disability Index (NDI) and the Copenhagen Neck Functional Disability Scale (CNFDS). Descriptive statistics were calculated for demographics and baseline questionnaire scores. To determine if the investigated sample sizes are equivalent, the chi-square test was used. The chi-square test was used to compare qualitative features between persons with scoliosis and healthy controls. In addition, a Mann-Whitney test was utilized to compare differences between both groups in regard to quantitative characteristics. To establish relations between quantitative data such as e.g. age, duration of brace application, apical translation, Cobb angle, and questionnaire results, we used Spearman's rank correlation (marked as rS). To determine dependency between quantitative and qualitative characteristics, e.g. between questionnaire numerical data and marital status, place of residence or curve type, ANOVA Kruskal-Wallis test was used. A *p<*0.05 indicates statistical significance. Statistical calculations were performed by Statistica software. In regards to RODI, RMQ, QDS, NDI and CNFDS (both for total scores and particular sub-sections), statistically significant differences (p <0.001) between both samples were found, indicating higher levels of pain and neck and lower back pain-related disability among persons with scoliosis. Associations exist between RODI and RMQ (rS = 0.76) QDS (rS = 0.70), NDI (rS = 0.69) and CNFDS (rS = 0.60). RMQ was associated with QDS (rS = 0.71) and NDI (rS = 0.69), whereas QDS correlated with NDI (rS = 0.80) and CNFDS (rS = 0.60). NDI was also associated with CNFDS (rS = 0.81). Persons with scoliosis treated in adolescence with a Milwaukee brace display significant restrictions in everyday activities, due to lower back pain (LBP) and neck-related disabilities, compared to healthy controls. In addition, back pain is associated with curve progression in long-term follow-up after conservative treatment. Moreover, LBP-related disability coexists with restrictions experienced due to neck pain.

## Introduction

Many researchers' results indicate that patients with adolescent idiopathic scoliosis, (AIS) suffer from back pain more frequently than healthy populations [1]. It was found that the incidence of back pain in patients with scoliosis treated conservatively was lower than that of the AIS study group (11% vs. 32%) [2–4].

It was revealed that the stabilization of scoliosis with a brace may decrease the likelihood of back pain induced by the altered scoliotic spinal mechanics, and presumably a failure to stabilize the spine (curve progression) may increase the incidence of spinal pain [5]. Additionally, some associations between types of orthoses and pain intensity in AIS patients have been discovered [5].

Considering the incidence of spinal pain in the long-term follow-up to treatment for AIS, most results refer to patients treated surgically [6–8]. For instance, Danielsson et al. [9] indicated that, more than 20 years after the completion of treatment with a brace, AIS patients had significantly more degenerative lumbar disc changes than the controls. They indicated that, even if conservatively-treated patients admitted to having pain more often than the control group, their pain was mostly mild, analgesics were rarely used, and no patient had a major functional deficit. Weinstein et al. [10] presented a 50-year follow-up of non-operatively treated late-onset persons with scoliosis. Overall, AIS patients were found to be “productive and functional at a high level” with some restraints due to back pain and cosmetic concerns. Meanwhile, Gabos et al. [11], in a study of long-term radiographic and functional outcomes of females with AIS who had completed a Wilmington orthosis treatment, found that there was no significant overall difference between the group who had undergone orthotic treatment and the control group in terms of back pain or physical, functional or personal care activities. However, patients reported significantly greater difficulty with selected positional activities [11]. However, it must be emphasized that these findings may relate either to the condition of scoliosis or the wearing of the brace and it is not possible to make such a distinction.

At the same time, very little has been written about problems in the cervical spine and neck pain-related difficulties in performing everyday activities in AIS patients, especially in a long-term follow-up after the completion of brace treatment. The first signs of a problem were described by Moskowitz et al. [12], who reviewed 61 patients who had had a posterior spine fusion for any type of scoliosis 25 years previously (range 20–30 yrs). An unexpectedly high incidence of cervical complaints (57%) was reported amongst those patients. Hilibrand et al. [13] published a radiographic study of the sagittal alignment of the cervical spine in patients with AIS. As this was a study only of alignment and only in adolescents, no comments could be made regarding adult pain problems or the assessment of cervical degeneration. Edgar and Mehta [14], in the only study involving the long-term follow-up of fused and unfused persons with scoliosis, found an incidence of cervicodorsal pain in 7.8% of the unfused patients and in 17.6% of the surgically treated patients.

Therefore, taking into account some inconsistencies between study results relating to back and neck pain in AIS patients after completing Milwaukee brace treatment and, in particular, the lack of comprehensive long-term assessments of cervical pain in adult persons with scoliosis, special emphasis should be placed on the following research questions: do adult persons with scoliosis treated with a Milwaukee brace during adolescence have more lower back pain (LBP) and neck pain compared with non-scoliotic subjects? How does spinal pain affect the daily life and the activities of these patients? Is there any correlation between the intensity of the pain, radiographic data and brace treatment-related variables? Thus, we aimed to provide a complex assessment of adult females with scoliosis treated with a Milwaukee brace, in a minimum 23-yrs follow-up. To sum up, the purpose of this report is to explore the long-term outcomes in terms of back and neck pain and functionality, in relation to radiological, clinical, pulmonary function and socio-demographic data.

Our hypothesis is that no difference is expected between study and control groups in terms of socio-demographic characteristics, pain level and neck- or low back pain-disability. In addition, we assumed that there will be a significant relationship between pain intensity, patients’ everyday activities and radiographic, clinical, pulmonary and brace treatment-related variables. All study aims have been achieved.

## Material and methods

### Study design

Results concerning back- and neck pain and function in consecutively selected adult AIS females (scoliosis group-SG) treated with a Milwaukee brace were evaluated. Based on an extensive search of Pediatric Orthopedics and Traumatology Clinic charts, we retrospectively reviewed the clinical records and radiographs of all female patients who had successfully completed a course of treatment with the Milwaukee orthosis between 1974 and 1990. In those patients, the Risser sign 4 and minimum two years post-menarche was defined as a maturity, after that time the brace treatment was completed. Forty patients met the criteria for inclusion, but due to change some personal details (such as address or family name after getting married), not all of them were contacted. Finally, 30 women returned for a follow-up evaluation. To make the obtained data more reliable, an age- and sex-matched control group of 42 healthy individuals (healthy controls group–HG) was selected and asked to complete the same questionnaires as the patient group.

### Study protocol

Study participants from both groups were examined using the same protocol, except for the radiological evaluation. The contents of interview in both study groups included age, work, marital status, number of children, incidence of caesarian sections and complications during delivery, educational level, place of residence and the pursuit of active hobbies. In addition, to evaluate the implications of the brace for back and neck pain and functionality, all study participants were asked to fill in the same series of questionnaires.

### Clinical and radiological examination

Clinical and radiological examinations were performed three times, before and after completing the treatment and then in the follow-up, and were taken in an upright position with the iliac ala exposed in an anterior-posterior projection. Data concerning former treatment regimens and radiological findings were gathered from reviews of charts and radiographs. The physical examinations were performed by AO, the 3^rd^ study author, and the radiographic measurements were conducted by JG and MG, the 2^nd^ and the 5^th^ study authors, respectively.

The success rate at maturity was calculated according to Nachemson and Peterson, who defined a successful treatment as an increase in the curve of less than 6° from the start of bracing [15]. The change in curvature from the end of treatment until the follow-up examination was also assessed. Pulmonary function, in terms of vital capacity (VC), was evaluated three times, before and after completing the treatment and then in the follow-up. The VC examinations at the present follow-up were performed by ML, the 4^th^ study author.

### Healthy controls

The exclusion criteria for the control group were as follows: previous back surgery or significant scoliosis, which was ruled out by clinical examination, including using Perdriolli’s scoliometer. None of the controls had a trunk rotation of more than 5°, according to Danielsson et al. [16].

### Patient sample

Thirty AIS patients, with a minimum of 23-yrs of follow-up after completing Milwaukee brace treatment, were included in the study. All treatments were completed before the patient reached 19 yrs of age. In all cases, scoliosis had not been detected before the age of 10 and was not combined with any major spinal deformities at the time when the brace treatment was implemented. In addition, patients were excluded from the study if they, at the time of the follow-up examinations, suffered from any other disease leading to trunk deformity. The investigated sample sizes are equivalent (p = 0.157).

During the whole treatment non-compliance monitoring such as temperature probe was not used. The average values were based on interviews with patients during the clinical examination. In addition, to make the obtained data more credible, daily duration of brace wearing was confirmed during the separate interviews carried out with parents.

### Ethical issues

All study participants were informed in detail about the objective of the study. They understood that they would be anonymous and that their personal information would not be disclosed. All participants signed written informed consent forms in order to participate in the study. The study was approved by the Bioethics Commission of Poznan University of Medical Sciencesand was carried out following universal ethical principles.

### Evaluation of neck and back pain-related disability

Polish versions of Revised Oswestry Low Back Pain Disability Index (RODI), Rolland-Morris Questionnaire (RMQ), Quebec Back Pain Disability Scale (QDS), Neck Disability Index (NDI) and Copenhagen Neck Functional Disability Scale (CNFDS) enabled us to assess the intensity of cervical and lumbar pain and the ability to perform everyday activities among both study samples [17–19].

*The RODI* is a revised version of the original Oswestry Disability Index [20] and focuses on, alongside the subjective evaluation of pain intensity, the degree to which everyday activities such as personal care, lifting, walking, sitting, rising/standing, sleep, social life, traveling and changes in pain intensity are affected. The answers are marked on a six-point scale (from 0 to 5), where 0 corresponds to no limitations on functional status, and 5 indicates maximum restrictions. The maximum sum of points from all the sections is 50. In order to present the general result in percentage values, reflecting the extent to which the ability to carry out everyday activities is restricted, the total is divided by 2 [20,21]. A result of 0–4 points is interpreted as no limitation to everyday activities, 5–14 points indicates mild limitation, 15–24 points is moderate limitation, 25–34 points indicates a serious disorder, and a score over 34 points is evidence of disability. [20,21]. The RODI's psychometric properties have been well established. The RODI correlates with other outcome measures aiming at measuring disability due to LBP [22–26]. The RODI shows good construct validity because it was used as the standard of comparison for other outcome measures assessing LBP-induced disability. Internal consistency has been shown to be at the acceptable level by different authors. Cronbach alpha ranges from 0.71 to 0.87 [24,25]. Test-retest reliability has been shown to be high. Values range from r *=* 0.83 to 0.99 and vary according to the time interval between measurements [22–25]. The longer the wait between repeated measure, the lower the score becomes. Intraclass correlation coefficient values from 0.84 to 0.94 have been reported [23]. Responsiveness has been reported to be high. The most common method for measuring responsiveness found in the current literature search was a receiver operating characteristic curve. It can also be used to estimate the minimum clinically important difference (MCID). Values for the area under the curve range from 0.723 to 0.94. The MCID has been reported to be between 4 and 10.5 points [23,24].

*The RMQ* is widely used to evaluate the degree of disability due to lumbosacral back pain [20]. It comprises 24 statements requiring a “yes”/”no” answer. The total score can range from 0 (no disability) to 24 (severe disability). The subjects were divided into the following four disability groups according to their scores: low degree of disability: 4–10 points, moderate: 11–17 and severe: 18–24. If the result was ≤ 3, the patient was considered to have no disability [27]. Test-retest reliability of RMQ was 0.91 [27], and Cronbach's alpha coefficients in outpatients with low back pain diagnosed with a musculoskeletal origin were 0.90, 0.84, 0.89, 0.92 [28]. Considering responsiveness in patients experiencing lower back pain, the Standard Response Mean was 0.55 (95% CI = -0.54 to 1.64) [29].

*The QDS* is comprised of 20 questions and 6 domains, reflecting everyday activities such as sleep/rest; sitting/rising; walking; moving; bending/squatting; lifting heavy objects. The responses are marked on a scale of 0–5, where 0 corresponds to no limitations, whereas 5 signifies maximum restrictions to everyday functional status. The overall result varies from 0 (no worsening of spine function) to 100 (maximum restrictions on functional status) [21, 30]. Test-retest reliability of QDS was 0.92, and Cronbach's alpha coefficient was 0.96. The scale correlated as expected with other measures of disability, pain, medical history, and utilization variables, work-related variables, and socio-demographic characteristics. Significant changes in disability over time, and differences in change scores between patients that were expected to differ in the direction of change were also fund [30].

*The NDI* questionnaire assesses pain intensity and related limitations to cervical spine function during everyday activities [31]. The NDI is comprised of 10 questions regarding: pain intensity, personal care, lifting, reading, headaches, concentration, work, driving, sleeping, and recreation [31]. Each item is scored from 0 (no disability) to 5 (total disability). The maximum possible score is 50. However, the total score obtained is often doubled to give a percentage score, out of 100. The interpretation is as follows: 0–20 normal, 21–40 mild disability, 41–60 moderate, 61–80 severe and <80 complete or exaggerated disability [31]. Test-retest reliability of NDI resulted in good statistical significance (Pearson's r = 0.89). The Cronbach alpha coefficients were calculated from a pool of questionnaires completed by 52 such subjects resulting in a total index alpha of 0.80, with all items having individual alpha scores above 0.75. Concerning concurrent validity, NDI scores were compared to scores on the McGill Pain Questionnaire, with similar moderately high correlations (0.69–0.70), whereas the responsiveness was excellent (effect size = 0.85) [32].

*The CNFDS* consists of 15 items that evaluate the impact of neck pain. Three items evaluate pain severity directly, including the patient’s perception of the future impact of neck pain, eight items evaluate disability during everyday activities and four items focus on social interaction and recreation [33]. There are three possible answers to select from each item; ‘‘yes” (2 points), ‘‘occasionally” (1 point), and ‘‘no” (0 points). For items 1–5, however, the scoring is reversed and here “yes” carries a score of 0, “occasionally” 1 and “no” 2. The highest score attainable is 30, indicating the worst possible impact, and the lowest is 0, where no impact of neck pain can be identified [33].

The Cronbach's alpha coefficient of the CNFDS for internal consistency was 0.9 for the entire scale, and the coefficients for individual items were all greater than 0.88. Disability scale scores correlated strongly to pain scores as well as to doctor and patient global assessments, indicating good construct validity [33]. Relative changes in disability scores demonstrated a moderately strong correlation to changes in pain scores after treatment. Scale scores correlated weakly to all physical measurements. The disability scale demonstrated excellent practicality and reliability. The scale accurately reflects patient perceptions regarding functional status and pain as well as doctor's global assessment and is responsive to change over long periods of time [33].

### Statistical analysis

For statistical quantitative (numerical) features, e.g. age, apical translation, Cobb angle, number of children or questionnaire results, we calculated the mean, a 95% confidence interval, the range and standard deviation (SD). With respect to qualitative features (information that has aspects that are impossible to be measured), e.g. curve type, educational level, marital status or place of residence, we gave the number of units that belong to the described categories of a given feature and respective percentages. To determine if the investigated sample sizes are equivalent, the chi-square test was used. The chi-square test was used to compare qualitative features between persons with scoliosis and healthy controls. In addition, a Mann-Whitney test was utilized to compare differences between both groups in regard to quantitative characteristics. To establish relations between quantitative data such as e.g. age, duration of brace application, apical translation, Cobb angle, and questionnaire results, we used Spearman's rank correlation (marked as rS). To determine dependency between quantitative and qualitative characteristics, e.g. between questionnaire numerical data and marital status, place of residence or curve type, ANOVA Kruskal-Wallis test was used. A *p<*0.05 indicates statistical significance. Statistical calculations were performed by Statistica software. See the supplementary material file containing clinical, radiological, socio-demographic and questionnaire data.

## Results

### Patient clinical and radiological data

Patients’ follow-up period was mean 27.77 yrs ± SD 3.30 (range 23–35). The Milwaukee brace was worn for a mean of 22.9 hrs daily ± SD 0.31 (range 22–23). The duration of treatment was mean 45.47 months ± SD 20.00 (range 24–104).

Following the criteria of the Scoliosis Research Society regarding the location of the apex [34], thoracic scoliosis was identified in 21 patients (70%), thoracolumbar in 2 patients (6.67%) and lumbar curves were identified in 7 AIS females (23.33%). Radiographic examinations at the beginning of brace the treatment resulted in Risser Grade 0 in 19 patients (63.33%), Risser Grade I in 2 patients (6.67%), and Risser Grade II in 9 patients (30%). Risser Grade IV was identified after completing treatment in all patients involved in the study (100%). The European Risser sign was used to define skeletal maturity status. The success rate at maturity, according to Nachemson and Peterson [15], was identified in 16 patients (53.33%). Five patients (16.67%) were eligible for scoliosis surgery after completing the brace treatment, but refused to undergo an operation. Change in spine curvature from the end of treatment until the follow-up was mean 9.1 angles ± SD 7.64 (range 0–27). Mean VC was 2526.67 cm^3^ ± SD 561.98 in the pretreatment evaluation, 2983.33 cm^3^ ± SD 617.05 after treatment and 2569.64 cm^3^ ± SD 591.41 in the final evaluation. For additional clinical and radiological characteristics of the patient sample, see Table 1.

**Table 1:** Clinical characteristics of patients.

Characteristics	Mean ± SD	Range	N (%)
Brace application [hours/day]	22.9 (0.31)	22–23	---
Brace application [months]	45.47 (20.00)	24–104	---
Follow-up after completing treatment [years]	27.77 (3.30)	23–35	---
Body Mass Index			---
Before treatment	17.47 (3.78)	13.64–22.83	---
After completing treatment	19.91 (2.57)	15.81–25.24	---
At most recent follow-up	24.03 (4.05)	17.78–34.34	---
Curve type			
Thoracic	---	---	21 (70.0)
Thoraco-lumbar	---	---	2 (6.67)
Lumbar	---	---	7 (23.33)
Curve size of the major curve (Cobb angle)			
Before treatment	32.2 (5.59)	20–40	---
After completing treatment	37.87 (12.75)	10–70	---
At follow-up	45.03 (17.41)	10–97	---
Success rate at maturity*	---	---	16 (53.33)
Curvature change from end of treatment to present	9.1 (7.64)	0–27	---
Apical translation [cm]**			
Before treatment	2.05 (0.98)	0.2–4	---
After completing treatment	2.65 (1.30)	0.3–5.1	---
At most recent follow-up	3.66 (1.99)	0.5–9.4	---
Rib hump angle at follow-up	10.37 (4.20)	4–20	---
Rib hump height at follow-up [cm]	3.33 (1.53)	1–7	---
Thoracic kyphosis at follow-up [angle]	25.33 (11.06)	9–62	---
Lumbar lordosis at follow-up [angle]	54.63 (10.59)	27–76	---
Vital capacity (VC) [cm^3^]			
Before treatment	2526.67 (561.98)	1500–3500	---
After completing treatment	2983.33 (617.05)	1600–4000	---
At most recent follow-up	2569.60 (591.41)	1340–3610	---

Note

*According to Nachemson and Peterson, successful treatment was defined as an increase in the curve of less than 6° from the start of bracing

** the degree of apical translation of the center sacral vertical line (CSVL) according to the Harms Study Group; standard deviation (Mean ± SD).

### Socio-demographic data

The age of the patients during the follow-up was mean 41.13 yrs ± SD 3.87 (range 35–55), whereas age of the controls was mean 42.05 yrs ± SD 7.41 (range 22–61). Twenty-eight females with AIS (93.4%) and 29 healthy controls (69%) were married. Of 30 female patients, 29 (96.67%) had children, and the number of children was mean 2.0 ± SD 0.83 (range 0–4), whereas in HC group 33 females (78.57%) had children, and the number of children in this subgroup was mean 1.48 ± SD 0.99 (range 0–3). The rate of cesarean section was 30% (9 patients) in SG and 27.3% (9 controls) in HG. Ten patients (34.48%) and 8 controls (23.53%) had experienced problems during delivery (for additional data, see Table 2).

**Table 2:** Socio-demographic characteristics of patients and healthy controls.

Characterisatics	Patient group			Healthy controls			p value
	Mean ± SD	Range	N (%)	Mean ± SD	Range	N (%)	
Age at the start of treatment [yrs]	12.43 (1.83)	10–14	---	Not applicable			
Age at the most recent follow-up [yrs]	41.13 (3.87)	35–50	---	42.05 (7.41)	22–61	----	p = 0.326
Marital status							p = 0.043*
Single	---	---	1 (3.33)	---	---	8 (19.0)	
Married	---	---	28 (93.34)	---	---	29 (69.00)	
Divorced	---	---	1 (3.33)	---	---	5 (11.9)	
Educational level							p = 0.008*
Elementary	---	---	2 (6.67)	---	---	2 (4.8)	
Occupational	---	---	7 (23.33)	---	---	0 (0)	
Secondary	---	---	7 (23.33)	---	---	19 (45.2)	
University	---	---	14 (46.67)	---	---	21 (50)	
Place of residence							p = 0.0028*
Country	---	---	12 (40.0)	---	---	8 (19.0)	
City below 25 000 of inhabitants	---	---	12 (40.0)	---	---	7 (16.7)	
City between 25 000 and 200 000 of inhabitants	---	---	2 (6.67)	---	---	4 (9.5)	
City over 200 000 of inhabitants	---	---	4 (13.33)	---	---	23 (54.8)	
Working time per week [hours]	26.93 ()	0–60	---	42.88 (13.96)	5–70	---	p = 0.013*
Overall working time [yrs]	17.37 (19.13)	0–30	---	19.55 (8.94)	1–40	---	p = 0.317
No. of children	2.0 (0.83)	0–4	---	1.48 (0.99)	0–3	---	p = 0.046*
Age at 1^st^ pregnancy**	24.86 (3.99)	19–38	---	24.50 (3.72)	18–34	---	p = 0.767
Caesarian section*	---	---	9 (30)	---	---	9 (27.3)	p = 0.745
Complications during delivery**	---	---	10 (34.48)	---	---	8 (23.53)	p = 0.344
Active hobby	---	---	13 (43.33)	---	---	16 (38.1)	p = 0.656
Active hobby per week [hours]***	3.46 (3.78)	1–15	---	5.12 (4.30)	2–20	---	p = 0.038*

Note

* p < 0.05

**out of 29 patients and 34 participants who had had a child

***out of 13 patients and 17 healthy controls participating in active hobbies; standard deviation (Mean ± SD)

### Low back pain and function

Regarding RODI, patients scored mean 13.67 ± SD 6.84 and healthy controls scored mean 5.26 ± SD 5.40. Regarding results expressed as percentage values, SG scored 27.34%, whereas HG scored 10.52%, which is interpreted as moderate and minimal disability respectively. In SG, 8 patients (26.67%) showed minimal disability, 16 subjects (53.33%) reported moderate disability and severe disability was indicated in 6 patients (20%), whereas 37 healthy controls (88.10%) reported minimal disability, and 4 and 1 participants, that is, 9.52% and 2.38% respectively, suffered moderate and severe restrictions in everyday activities.

The value of the RMQ was mean 5.60 ± SD 4.68 in the SG, indicating a low degree of disability, and mean 1.79 ± SD 2.35 in HG, meaning no disability. In SG 12 patients (40%) reported no disability, 13 subjects (43.34%) reported low degree of disability, moderate disability was indicated in 4 patients (13.33%), and severe disability was identified in 1 patient (3.33%). 26 healthy controls (85.72%) reported no disability, and 5 and 1 participants, or 11.90% and 2.38% respectively, revealed low and moderate levels of restriction. The mean QDS-PL scores were 23.28 Mean ± SD 14.53 in SG and 8.23 Mean ± SD 7.79 in HG. Considering the results for particular criteria, the highest scores, relating to the most severe limitations, are related to moving and leaning/squatting in both study samples (mean 5.13 ± SD 2.92, mean 4.24 ± SD 3.19 in SG and mean 1.81 ± SD 1.69, mean 1.57 ± SD 1.84 in HG respectively).

### Cervical pain and functionality

The value of the NDI general result equaled mean 11.66 ± SD 7.43 in SG and mean 4.38 ± SD 4.02 in HG. Considering results expressed as percentage values, SG scored 23.32%, whereas HG scored 8.76%, which is interpreted as mild and no disability respectively.

In SG 5 patients (16.67%) reported no disability, 15 subjects (50%) reported mild disability, moderate disability was indicated in 8 patients (26.67%) and 2 patients (6.67%) demonstrated severe restrictions to their everyday activities, whereas 27 healthy controls (64.29%) reported no disability, and 14 and 1 participants, or 33.33% and 2.38% respectively, revealed mild and moderate levels of disability.

Concerning the CNPDS total score, patients scored mean 12.63 ± SD 3.48, whereas the value in the healthy control sample was mean 2.14 ± SD 3.48. The highest values among both SG and HG concern the disability subscale (mean 8.17 ± SD 4.2.57 and mean 1.24 ± SD 1.75 respectively).

### Comparative analyses

Among a variety of socio-demographic variables which might be affected by an individual’s spinal function, significant differences could be found between the groups such as marital status (p = 0.043; patients from SG got married more often), educational level (p = 0.008; patients from SG achieved lower levels of education), place of residence (p = 0.028, patients from SG lived more often in smaller town and cities), working time per week (p = 0.013, patients from SG spent less time each week on work-related activities), no. of children (p = 0.046, patients from SG had more children) and time spent on active hobbies per week (p = 0.038, patients from SG spend less hrs each week on active hobbies). For details, see Table 2.

It was also revealed, regarding RODI, RMQ, QDS, NDI and CNFDS (both for total scores and individual domains), statistically significant differences (p <0.001), between both samples, have been confirmed, indicating higher levels of pain, and neck and lower back pain-related disability among persons with scoliosis. For details see Table 3.

**Table 3:** Descriptive statistics of the RODI, RMQ, QDS, NDI and CNFDS results.

Questionnaire	Patients						Healthy controls						p value
	Mean	95% CI		Min	Max	Mean ± SD	Mean	95% CI		Min	Max	Mean ± SD	
		from	to					from	to				
RODI-Total score	13.67	11.11	16.22	0	30.0	6.84	5.26	3.58	6.95	0	25.0	5.40	p < 0.001*
RMQ	5.60	3.85	7.35	0	18.0	4.68	1.79	1.05	2.52	0	12.0	2.35	p < 0.001*
QDS- Total score	23.28	17.75	28.80	0	62.0	14.53	8.26	5.83	10.69	0	41.0	7.79	p < 0.001*
Sleeping/resting	2.87	1.95	3.79	0	9.0	2.46	0.95	0.61	1.30	0	5.0	1.10	p < 0.001*
Sitting/Rising	3.03	2.18	3.89	0	10.0	2.30	1.31	0.92	1.70	0	5.0	1.26	p < 0.001*
Walking	3.97	2.79	5.15	0	11.0	3.16	1.24	0.73	1.75	0	7.0	1.64	p < 0.001*
Moving	5.13	4.04	6.22	0	11.0	2.92	1.81	1.28	2.33	0	8.0	1.69	p < 0.001*
Leaning/squatting	4.24	3.03	5.46	0	11.0	3.19	1.57	1.00	2.14	0	8.0	1.84	p < 0.001*
Lifting	3.47	2.38	4.56	0	12.0	2.92	1.38	0.83	1.93	0	9.0	1.75	p < 0.001*
NDI-Total score	11.66	8.83	14.48	2.0	27.0	7.43	4.38	3.12	5.63	0	20.0	4.02	p < 0.001*
CNPDS-Total score	12.63	10.73	14.53	7.0	25.0	5.09	2.14	1.06	3.23	0	15	3.48	p < 0.001*
Pain severity	2.63	2.21	3.05	0	5.0	1.13	0.48	0.17	0.79	0	4.0	0.99	p < 0.001*
Social interaction	1.83	1.02	2.64	0	7.0	2.17	0.43	0.03	0.82	0	6.0	1.27	p < 0.001*
Disability	8.17	7.21	9.13	4	13.0	2.57	1.24	0.69	1.79	0	6.0	1.75	p < 0.001*

Note

* p < 0.05; Revised Oswestry Low Back Pain Disability Index (RODI); Rolland-Morris Questionnaire (RMQ); Quebec Back Pain Disability Scale (QDS); Neck Disability Index (NDI); Copenhagen Neck Functional Disability Scale (CNFDS); standard deviation (Mean ± SD); confidence intervals (CI).

In particular, regarding RODI, significant differences concerning personal care, lifting, walking, sitting, rising/standing, social life, traveling and changes in pain intensity (p < 0.001), except for sleeping (p = 0.099) were confirmed. For details see Table 3.

### Associations between pain, functionality, clinical and radiological factors

Considering lower back pain-related physical impairment, we identified significant associations between RMQ and BMI before treatment (rS = -0.44) and Cobb angle after completing the treatment (rS = 0.36). We also identified significant associations between particular subscales of QDS (moving and BMI before treatment (rS = -0.42)), VC before treatment (rS = -0.39), between lifting and Cobb angle during follow-up examinations (rS = 0.38) and between total score and apical translation after completing the treatment (rS = 0.38) and thoracic kyphosis during the follow-up (rS = 0.39) (see Table 4 and Table 5).

**Table 4:** Correlational analysis between clinical and radiological patient characteristics and RODI, RMQ, NDI and CNFDS.

Characteristics	RODI-Total score	RMQ	NDI-total score	CNPDS			
Characteristics				Total score	Pain severity	Social interaction	Disability
Brace application [hours/day]	Not applicable						
Brace application [months]	rS = 0.13	rS = 0.21	rS = 0.13	rS = -0.08	rS = -0.21	rS = 0.05	rS = -0.03
Follow-up after completing treatment [years]	rS = -0.19	rS = 0.04	rS = -0.10	rS = 0.05	rS = 0.16	rS = -0.05	rS = 0.08
Body Mass Index							
Before treatment	rS = -0.32	rS = -0.44*	rS = -0.28	rS = 0.19	rS = 0.23	rS = 0.18	rS = 0.17
After completing treatment	rS = -0.30	rS = -0.28	rS = -0.19	rS = 0.22	rS = 0.19	rS = 0.29	rS = 0.24
At most recent follow-up	rS = -0,18	rS = -0.06	rS = -0,07	rS = -0.03	rS = 0.02	rS = 0.06	rS = 0.01
Curve type	p = 0.980	p = 0.710	p = 0.271	p = 0.482	p = 0.430	p = 0.614	p = 0.411
Curve size of the major curve (Cobb angle)							
Before treatment	rS = 0.03	rS = 0.01	rS = 0.12	rS = -0.07	rS = -0.10	rS = -0.05	rS = -0.03
After completing treatment	rS = 0.33	rS = 0.36*	rS = 0.15	rS = -0.09	rS = -0.07	rS = -0.06	rS = -0.09
At most recent follow-up	rS = 0.31	rS = 0.31	rS = 0.14	rS = -0.11	rS = -0.15	rS = -0.07	rS = -0.12
Curve change from end of treatment to most recent follow up**	rS = 0.01	rS = -0.02	rS = -0.10	rS = 0.11	rS = -0.02	rS = 0.15	rS = 0.08
Apical translation [cm]***							
Before treatment	rS = 0.08	rS = 0.12	rS = 0.12	rS = -0.11	rS = -0.11	rS = -0.17	rS = -0.08
After completing treatment	rS = 0.10	rS = 0.24	rS = 0.27	rS = -0.23	rS = -0.15	rS = -0.12	rS = -0.26
At most recent follow-up	rS = 0.31	rS = 0.23	rS = 0.05	rS = -0.18	rS = -0.13	rS = -0.16	rS = -0.19
Rib hump angle at most recent follow-up	rS = -0.09	rS = -0.04	rS = -0.15	rS = 0.11	rS = 0.15	rS = 0.15	rS = 0.06
Rib hump height at most recent follow-up [cm]	rS = -0.02	rS = 0.01	rS = -0.06	rS = -0.14	rS = -0.09	rS = -0.12	rS = -0.18
Thoracic kyphosis at most recent follow-up [angle]	rS = -0.03	rS = 0.02	rS = 0.16	rS = -0.10	rS = -0.06	rS = 0.03	rS = -0.12
Lumbar lordosis at most recent follow-up [angle]	rS = -0.01	rS = 0.06	rS = -0.10	rS = 0.09	rS = 0.07	rS = -0.05	rS = 0.11
Vital capacity (VC) [cm^3^]							
Before treatment	rS = -0.17	rS = -0.29	rS = -0.27	rS = 0.14	rS = 0.18	rS = 0.11	rS = 0.13
After completing treatment	rS = -0.02	rS = -0.10	rS = -0.13	rS = 0.14	rS = 0.09	rS = 0.11	rS = 0.16
At most recent follow-up	rS = -0.07	rS = -0.23	rS = -0.04	rS = -0.11	rS = -0.06	rS = -0.12	rS = 0.10

Note

* p < 0.05

**according to Nachemson and Peterson, successful treatment was defined as an increase in curvature of less than 6° from the start of bracing

*** the degree of apical translation of the center sacral vertical line (CSVL) according to the Harms Study Group; Revised Oswestry Low Back Pain Disability Index (RODI); Rolland-Morris Questionnaire (RMQ); Neck Disability Index (NDI); Copenhagen Neck Functional Disability Scale (CNFDS).

**Table 5:** Correlational analysis between clinical and radiological patients characteristics and QDS.

Characteristics	QDS							
	Total score	Sleeping/ resting		Sitting/ Rising	Walking	Moving	Leaning/ squatting	Lifting
Brace application [hours/day]	Not applicable							
Brace application [months]	rS = 0.01		rS = 0.06	rS = -0.10	rS = -0.07	rS = 0.09	rS = 0.10	rS = -0.14
Follow-up after completing treatment [years]	rS = 0.06		rS = 0.01	rS = 0.07	rS = 0.05	rS = -0.11	rS = -0.08	rS = 0.06
Body Mass Index								
Before treatment	rS = -0.17		rS = -0.06	rS = -0.13	rS = -0.06	rS = -0.42*	rS = -0.19	rS = -0.09
After completing treatment	rS = -0.03		rS = 0.14	rS = -0.09	rS = -0.17	rS = -0.16	rS = -0.03	rS = -0.14
At most recent follow-up	rS = 0.14		rS = 0.14	rS = -0.07	rS = -0.01	rS = -0.10	rS = 0.12	rS = 0.02
Curve type	p = 0.542		p = 0.096	p = 0.630	p = 0.584	p = 0.249	p = 0.730	p = 0.776
Curve size of the major curve (Cobb angle)								
Before treatment	rS = 0.29		rS = 0.20	rS = 0.24	rS = 0.20	rS = -0.04	rS = 0.24	rS = 0.12
After completing treatment	rS = 0.32		rS = 0.25	rS = 0.19	rS = 0.27	rS = 0.07	rS = 0.31	rS = 0.30
At most recent follow-up	rS = 0.27		rS = 0.29	rS = 0.20	rS = 0.33	rS = 0.03	rS = 0.32	rS = 0.38*
Curve change from end of treatment to present follow up**	rS = -0.09		rS = 0.07	rS = -0.02	rS = 0.05	rS = -0.07	rS = -0.08	rS = 0.15
Apical translation [cm]***								
Before treatment	rS = 0.19		rS = 0.12	rS = 0.19	rS = 0.21	rS = -0.16	rS = 0.22	rS = 0.12
After completing treatment	rS = 0.38*		rS = 0.26	rS = 0.30	rS = 0.34	rS = 0.52	rS = 0.32	rS = 0.22
At most recent follow-up	rS = 0.12		rS = 0.07	rS = 0.07	rS = 0.16	rS = -0.04	rS = 0.11	rS = 0.25
Rib hump angle at most recent follow-up	rS = 0.17		rS = 0.16	rS = 0.26	rS = 0.13	rS = 0.01	rS = 0.09	rS = 0.18
Rib hump height at most recent follow-up [cm]	rS = 0.24		rS = 0.11	rS = 0.31	rS = 0.25	rS = 0.78	rS = 0.09	rS = 0.24
Thoracic kyphosis at most recent follow-up [angle]	rS = 0.39*		rS = 0.27	rS = 0.36	rS = 0.16	rS = 0.28	rS = 0.15	rS = 0.15
Lumbar lordosis at most recent follow-up [angle]	rS = 0.02		rS = 0.05	rS = 0.17	rS = 0.12	rS = -0.10	rS = -0.14	rS = 0.09
Vital capacity (VC) [cm^3^]								
Before treatment	rS = -0.21		rS = -0.20	rS = -0.06	rS = -0.06	rS = -0.39*	rS = -0.11	rS = 0.07
After completing treatment	rS = -0.17		rS = -0.06	rS = -0.01	rS = -0.11	rS = -0.92	rS = -0.03	rS = -0.03
At most recent follow-up	rS = -0.18		rS = -0.09	rS = -0.20	rS = -0.10	rS = -0.15	rS = -0.15	rS = -0.21

Note

* p < 0.05

**according to Nachemson and Peterson, success of treatment was defined as an increase in the curve of less than 6° from the start of bracing

*** the degree of the apical translation of center sacral vertical line (CSVL) according to the Harms Study Group; Quebec Back Pain Disability Scale (QDS).

### Correlational analysis by means of socio-demographic characteristics

Considering neck pain, a significant correlation was discovered between the level of education and CNPDS total score (patients with secondary education declared lower neck pain-related disability than patients with a university education, p = 0.035). Moreover, the level of education was found to be associated with pain severity, social interaction and disability (patients with an occupational education reported lower neck pain-related disability than patients with a university education, p = 0.012, p = 0.028 and p = 0.039, respectively). For details see Table 6.

**Table 6:** Associations between the socio-demographic data and questionnaire results.

Questionnaires	Age at the present [yrs]	Age at the start of treatment[yrs]	Marital status	Educational level	Place of residence	Working time per week [hours]	Overall working time [yrs]	No. of children	Active hobby per week [hours]
RODI-Total score	rS = -0.13	rS = -0.27	Not applicable	p = 0.102	p = 0.173	rS = 0.13	rS = -0.09	rS = 0,14	rS = 0.09
RMQ	rS = 0.12	rS = -0.32		p = 0.232	p = 0.190	rS = 0.04	rS = -0.17	rS = -0.10	rS = 0.08
QDS- Total score	rS = 0.13	rS = 0.02		p = 0.062	p = 0.094	rS = 0.01	rS = -0.04	rS = 0.20	rS = 0.38
Sleeping/resting	rS = -0.03	rS = -0.24		p = 0.056	p = 0.040*	rS = 0.02	rS = 0.02	rS = 0.29	rS = 0.28
Sitting/Rising	rS = 0.07	rS = 0.17		p = 0.068	p = 0.341	rS = 0.03	rS = 0.01	rS = 0.21	rS = 0.28
Walking	rS = 0.02	rS = -0.05		p = 0.037*	p = 0.161	rS = -0.06	rS = 0.05	rS = 0.29	rS = 0.52
Moving	rS = 0.09	rS = -0.12		p = 0.126	p = 0.528	rS = 0.04	rS = -0.03	rS = 0.17	rS = 0.11
Leaning/squatting	rS = 0.02	rS = -0.01		p = 0.186	p = 0.095	rS = -0.01	rS = -0.01	rS = 0.10	rS = 0.09
Lifting	rS = 0.07	rS = 0.10		p = 0.174	p = 0.178	rS = 0.10	rS = 0.06	rS = -0.08	rS = 0.57*
NDI-Total score	rS = -0.08	rS = -0.06		p = 0.132	p = 0.380	rS = -0.03	rS = -0.20	rS = 0.15	rS = -0.06
CNPDS-Total score	rS = 0.05	rS = 0.23		p = 0.035*	p = 0.395	rS = 0.24	rS = 0.04	rS = 0.06	rS = 0.44
Pain severity	rS = 0.14	rS = 0.02		p = 0.012*	p = 0.289	rS = 0.22	rS = -0.05	rS = -0.05	rS = 0.66
Social interaction	rS = 0.02	rS = 0.09		p = 0.028*	p = 0.079	rS = 0.26	rS = 0.05	rS = -0.02	rS = 0.81
Disability	rS = 0.07	rS = 0.01		p = 0.039*	p = 0.358	rS = 0.23	rS = 0.05	rS = 0.08	rS = 0.32

Note

* p < 0.05

Revised Oswestry Low Back Pain Disability Index (RODI); Rolland-Morris Questionnaire (RMQ); Quebec Back Pain Disability Scale (QDS); Neck Disability Index (NDI); Copenhagen Neck Functional Disability Scale (CNFDS)

### Associations between neck and low back pain-related disability

As seen in Table 7, all total scores of RODI, RMQ, QDS, NDI and CNFDS display a significant intercorrelation. The strongest associations, above rS = 0.50, regard relationships between RODI and RMQ (rS = 0.76) QDS (rS = 0.70), NDI (rS = 0.69) and CNFDS (rS = 0.60). RMQ was associated with QDS (rS = 0.71) and NDI (rS = 0.69), whereas QDS correlated with NDI (rS = 0.80) and CNFDS (rS = 0.60). NDI was also associated with CNFDS (rS = 0.81). For details results see Table 7.

**Table 7:** Associations between total scores of RODI, RMQ, QDS, NDI and CNFDS.

Questionnaires	RODI	RMQ	QDS	NDI	CNPDS
RODI	---	rS = 0.76*	rS = 0.70*	rS = 0.69*	rS = 0.60*
RMQ	rS = 0.76*	---	rS = 0.71*	rS = 0.69*	rS = 0.40*
QDS	rS = 0.70*	rS = 0.71*	---	rS = 0.80*	rS = 0.60*
NDI	rS = 0.69*	rS = 0.69*	rS = 0.80*	---	rS = 0.81*
CNPDS	rS = 0.60*	rS = -0.40*	rS = 0.60*	rS = 0.81*	---

Note

* p < 0.05

Revised Oswestry Low Back Pain Disability Index (RODI); Rolland-Morris Questionnaire (RMQ); Quebec Back Pain Disability Scale (QDS); Neck Disability Index (NDI); Copenhagen Neck Functional Disability Scale (CNFDS).

## Discussion

The aim of this report was to elucidate the long-term outcomes of back and neck pain and functionality in a group of patients treated with a Milwaukee brace. The Milwaukee brace has been a standard of nonsurgical treatment for scoliosis since 1954. The Milwaukee brace, which should be worn for 23 hours every day, is most effective for the treatment of adolescent idiopathic scoliosis. Spinal deformity is corrected with the Milwaukee brace, in theory, by both passive and active forces. Passive correction is achieved by direct pressure from the pads or by traction from the brace design. Active correction is believed to occur through active movement of the body away from pressure points, as the patient’s muscles acted to pull the trunk away from contact with the lateral pads or chin support [35,36].

The Milwaukee brace remains the orthosis with the longest clinical history and the highest reported success rate in halting the progression of AIS, with the caveat that, because of the prior lack of standardization in bracing studies, direct comparison with studies of alternative brace designs is problematic [37,38]. Currently, the Milwaukee brace is primarily prescribed for patients with thoracic apices above Th7, for control of upper thoracic sagittal deformities, and for other spinal deformities not amenable to treatment with lower-profile designs [29]. A number of long-term follow-up studies on AIS have been published [12,39–43]. However, most of them refer to patients treated surgically [6,7,44], with no comparison group of healthy controls [43]. Furthermore, to date, assessment of spinal pain in persons with scoliosis in long-term evaluations has been mainly limited to LBP-related disability. To our knowledge, this is the first study of patients at least 23 years after the completion of Milwaukee brace treatment, involving a detailed evaluation of cervical pain and disability influencing everyday activities. A control group of randomly-selected healthy females was also included for comparative purposes.

Several authors have claimed that idiopathic scoliosis is not associated with a higher incidence of back pain [45]. For example, as mentioned above, Gabos et al. [11] indicated that there was no significant overall difference between the group who had undergone orthotic treatment and the control group in terms of back pain, physical, functional and self-care activities.

Lange et al. [46] evaluated the long-term outcome in AIS patients 12 years or more after treatment with the Boston brace. 12% of patients reported that they had consulted a physician for back complaints during the last year before follow-up, and 28% had had physiotherapy. Overall, back function was considered excellent, good or fair in 95% of the patients [46].

Haefeli et al. [47] in a retrospective study on patients 10 to 60 years of age after nonoperative treatment for AIS investigated long-term outcome regarding pain, disability, psychological disturbance, and health-related quality of life (HRQOL). Although pain, disability, HRQOL, and general psychological well-being were found to be quite satisfactory, curve size was found to be a significant predictor of pain in the long term. Danielsson and Hallerman [48] investigated consecutively-selected patients with idiopathic scoliosis, who were invited to a clinical follow-up at least 10 years after treatment with a brace or surgery. Overall, 77% reported back pain, but analgesic use was rare and 88% had normal back function as measured by the ODI. They concluded that most braced and surgically treated patients had a normal quality of life and that their physical functionality was only mildly impaired. Despite frequent back pain, back function was not severely affected. In the examined group of AIS patients, back pain and pain-related disability was a significant problem when compared to healthy controls, which confirms our primary hypothesis. The difference is statistically significant at p < 0.001 for RODI, RMQ and QDS, both in terms of total score and certain everyday activities such as walking, moving, lifting or leaning/squatting. Moreover, according to RODI, 73.33% of patients reported a moderate or severe degree of disability, whereas, in line with the RMQ criteria, moderate or severe disability was reported by just 16.63% of respondents. However, mean levels of disability, according to RMQ, are still higher among SG. In addition, according to QDS subscales, the highest levels of disability involved difficulties with activities such as moving and leaning/squatting. Those results are contrary to conclusions drawn by the Danielsson and Hallerman, Lange et al. or Danielsson and Nachemson studies [9,46,48].

Considering the associations between LBP-related disability and clinical and radiological factors, Danielsson and Nachemson indicated that pain and reduced back function was not related to a variety of curve-related factors, including degenerative lumbar disc changes. This lack of correlation between curve and functional outcome scores have been previously reported [9]. Ramirez et al. [5] indicated that back pain after brace treatment for AIS correlated with a family history of scoliosis, sport activities, and with curve progression during treatment, with the latter being the most significant. Patients with painful scoliosis which progresses during the bracing should be fully evaluated for an underlying non-idiopathic cause, but curve progression is the most likely explanation for the development of pain. The incidence of pain is significantly increased in patients with a family history of scoliosis or in patients engaged in sport activities. According to Ramirez et al. [5] pain incidence was found not to correlate with age at presentation, age at brace initiation, length of follow-up, gender, menarchal status, and limb-length discrepancy. Curve magnitude at presentation or at the time of brace prescription and skeletal maturity also did not show any correlation with symptoms [5]. Interestingly, our study result confirmed significant associations between RMQ and BMI before treatment, Cobb angle after completing the treatment, and between particular subscales of QDS: moving and BMI before treatment, VC before treatment, between lifting and Cobb angle at follow-up examinations and between total score and apical translation after completing the treatment and thoracic kyphosis at follow-up examinations. This indicates that back pain is associated, among others, with curve progression in a long follow-up after Milwaukee brace treatment. However, those results may relate either to the condition of scoliosis or the wearing of the brace and it is not possible to make such a distinction.

As previously stated, very little has been said on neck pain and cervical pain-related restrictions in performing everyday activities, such as social interaction and recreational activities, especially in long-term follow-up after the completion of brace treatment. As previously stated, Moskowitz et al. [12] revealed an unexpectedly high incidence of cervical complaints (57%) amongst AIS patients treated surgically in a 25-years follow-up, whereas Edgar and Mehta [14] found an incidence of cervicodorsal pain in 7.8% of the unfused persons with scoliosis and in 17.6% of the surgically treated patients. Interestingly, results derived from the current study confirm our primary hypothesis and support results derived from previous research conducted by e.g. Moskovitz et al or Mehta et al. [12,14] Regarding NDI results, most patients (83.34%) report mild, moderate or severe disability, whereas most of the controls (64.29%) reported no disability. Referring to CNPDS scores, SG revealed higher levels of restrictions regarding the function of the cervical spine, regarding pain level, disability and social interactions. Moreover, neck pain-related disability is not, unlike LBP-related restrictions, associated with current or past deformity-related scoliosis parameters, such Cobb angle or apical translation.

Furthermore, some interesting associations discovered between the level of education and CNPDS must be discussed. It was revealed that patients with university education declared higher levels of neck pain-related disability than patients with lower levels of education. Those results are in accordance with several cross-sectional studies which also have found a positive association between the duration of occupational sitting and occurrence of pain in the neck–shoulder region [49–51], while prospective studies found associations between sitting and neck–shoulder pain. It is well documented that white-collar workers spend a substantial proportion of their time at work sitting [52,53]. Thus, investigations of associations between sitting and neck–shoulder disorders are often conducted on workers in what is usually considered “sedentary” occupations, such as office-based jobs [50,54].

In addition, concerning associations between all study questionnaires, we identified intercorrelations between neck and lower back pain-related disability. Bearing in mind the advantages and disadvantages of the correlational study design, we cannot provide a conclusive reason as to why this relationship exists, but at the same time we are able to make some predictions e.g. about the incidence of neck pain, based on the LBP already reported by AIS patients.

### Study limitations

Some limitations of the current study must be pointed out. Firstly, a long-term assessment of conservative treatment of AIS is necessarily retrospective, since the tools specific to LBP- and neck pain-related disability assessment, such as RODI or NDI, were not available at the time when the conservative treatment was implemented. Secondly, the response rate (n = 30) was relatively low. Thirdly, the current study specifically investigated female patients only, which may have had an impact on the distribution of scores and limit the scope of the findings. Fourthly, brace compliance monitoring was a speculation and lastly, as indicated above, a correlational study cannot be used to draw conclusions about the causal relationships between selected variables. To sum up, we cannot determine which is the cause and which is the effect in the relationships between e.g. neck- and LBP-related disability, which are significantly intercorrelated.

### Clinical and future research implications

Despite the aforementioned limitations, we can provide clinicians with reliable data concerning the incidence of lumbar and cervical pain and disability in the long-term follow-up after completing a Milwaukee brace treatment. In addition, we believe long-term follow-up studies can provide reliable information to patients who will undergo conservative treatment for AIS. Further investigations, with a longer follow-up and in a group of patients treated surgically for comparative purposes, are needed to improve upon the results of our research.

### Conclusions

This retrospective examination of the function of the lumbar and cervical spine, in a highly specific subset of patients, revealed that persons with scoliosis treated in adolescence with a Milwaukee brace display significant limitations in everyday activities, due to LBP- and neck-related impairment. In addition, back pain is associated with curve progression in a long follow-up after conservative treatment. Moreover, LBP-related disability coexists with restrictions experienced due to neck pain.

## Supporting information

S1 File Study participants data and RODI, RMQ, NDI and CNFDS. (XLS)
